# Is adjuvant chemotherapy necessary in older patients with breast cancer?

**DOI:** 10.1007/s12282-021-01329-7

**Published:** 2022-01-15

**Authors:** Midori Morita, Akihiko Shimomura, Emi Tokuda, Yoshiya Horimoto, Yukino Kawamura, Yumiko Ishizuka, Katsutoshi Sekine, Sayaka Obayashi, Yuki Kojima, Yukari Uemura, Toru Higuchi

**Affiliations:** 1 Division of Endocrine and Breast Surgery, Kyoto Prefectural University of Medicine, Kyoto, Japan; 2 Department of Surgery, Saiseikai Shiga Hospital, Shiga, Japan; 3 Department of Breast and Medical Oncology, National Center for Global Health and Medicine, 1-21-1 Toyama, Shinjuku-ku, Tokyo, 162-8655 Japan; 4 Department of Medical Oncology, National Cancer Center Hospital, Tokyo, Japan; 5 Department of Medical Oncology, Fukushima Medical University, Fukushima, Japan; 6 Department of Breast Oncology, Juntendo University, Tokyo, Japan; 7 National Center for Global Health and Medicine Research Course in Advanced Medical Specialties, Juntendo University Cooperative Graduate School, Tokyo, Japan; 8 Medical Oncology Center, Saitama City Hospital, Saitama, Japan; 9 Department of General Surgical Science, Gunma University, Maebashi, Japan; 10 Biostatistics Section, Department of Data Science, Center of Clinical Sciences, National Center for Global Health and Medicine, Tokyo, Japan; 11 Breast Surgery Unit, Japanese Red Cross Saitama Hospital, Saitama, Japan

**Keywords:** Adjuvant chemotherapy, Breast cancer, Japanese, Older patients

## Abstract

**Background:**

Due to the lack of clinical trials on the efficacy of chemotherapy in older patients, an optimal treatment strategy has not been developed. We investigated whether adjuvant chemotherapy could improve the survival of older patients with breast cancer in Japan.

**Methods:**

We retrospectively analyzed data of patients with breast cancer aged ≥ 70 years who underwent breast cancer surgery in eight hospitals between 2008 and 2013. Clinical treatment and follow-up data were obtained from the patients’ medical electric records.

**Results:**

A total of 1095 patients were enrolled, of which 905 were included in the initial non-matched analysis. The median age and follow-up period were 75 (range 70–93) and 6.3 years, respectively. Of these patients, 127 (14%) received adjuvant chemotherapy (Chemo group) while the remaining 778 (86%) did not (Control group). The Chemo group was younger (mean age in years 73 vs 76; *P* < 0.0001), had a larger pathological tumor size (mean mm 25.9 vs 19.9; *P* < 0.0001), and more metastatic axillary lymph nodes (mean numbers 2.7 vs 0.7; *P* < 0.0001) than the Control group. The disease-free survival (DFS) and overall survival (OS) did not differ significantly between the two groups (*P* = 0.783 and *P* = 0.558). After matched analyses, DFS was found to be significantly prolonged with adjuvant chemotherapy (*P* = 0.037); however, OS difference in the matched cohort was not statistically significant (*P* = 0.333).

**Conclusion:**

The results showed that adjuvant chemotherapy was associated with a reduced risk of recurrence, but survival benefits were limited.

**Supplementary Information:**

The online version contains supplementary material available at 10.1007/s12282-021-01329-7.

## Introduction

Breast cancer is the most commonly diagnosed cancer and the leading cause of cancer death worldwide [1]. More than two million women are diagnosed with breast cancer annually. The life expectancy as well as the number of older patients with breast cancer has increased [2]. Japanese women have the highest life expectancy globally, at 86.94 years [3], and the ratio of the older to the younger population has also increased globally. It is expected that the number of older patients with breast cancer will continue to increase [1], and there will be more occurrences that may need to be examined in daily practice.

Numerous clinical trials have been conducted that have established adjuvant chemotherapeutic strategies for early-stage breast cancer. However, older patients have been excluded from many clinical trials due to their comorbidities and deteriorated organ functions [4]; thus, these trials do not reliably assess the fitness of older patients for this treatment regimen. Particularly, there is less evidence about the effectiveness of chemotherapy, such as anthracyclines and taxanes, in patients aged ≥ 70 years [5]. The current National Comprehensive Cancer Network (NCCN) guidelines for breast cancer recommend that the guidelines should not be applied similarly to older patients and young patients [6]. The American Society of Clinical Oncology (ASCO) has proposed developing recommendations to improve the evidence for elderly patients in response to a critical need [7].

It is unclear whether the results of prior clinical trials apply to older patients since they are mostly excluded from clinical trials due to their comorbidities. It is, therefore, urgent important to investigate the validity of chemotherapy for older patients and avoid dispensable chemotherapy. This study aimed to explore the impact of adjuvant chemotherapy in older patients with breast cancer, aged ≥ 70 years who had undergone breast and axillary surgery in Japan.

## Methods

### Study design and patients

This retrospective cohort study was conducted in seven institutions: National Cancer Center Hospital, Juntendo University, Gunma University, Kyoto Prefectural University of Medicine, Fukushima Medical University, Japanese Red Cross Saitama Hospital, and Saiseikai Shiga Hospital. Patients aged ≥ 70 years who underwent breast cancer surgery in eight hospitals between January 2008 and December 2013 were enrolled. Patients who were diagnosed with non-invasive breast cancer, had neoadjuvant chemotherapy or were given only trastuzumab, had bilateral breast cancer, and who lacked clinical data were excluded. Clinical treatment, follow-up data, and baseline data including patient characteristics, cancer stage, tumor histologic characteristics, performance status (PS), were obtained from the patients’ medical records. This study was approved by the institutional review board of each hospital. The need for written informed consent was waived because of the retrospective nature of the study. We present the findings following the format recommended by the strengthening the reporting of observational studies in epidemiology (STROBE) guidelines.

### Outcomes

The primary outcome of this study was the overall survival (OS) and the secondary outcome was the disease-free survival (DFS). The follow-up period was from the date of surgery to the 31st of December 2019.

### Statistical analyses

Fisher’s exact test and the *χ*^2^ test were used in the analysis. To identify independent prognostic factors that could affect OS or DFS, we used univariate Cox proportional hazards regression models. The prognostic effect of the adjuvant chemotherapy was examined using multivariate Cox regression analyses, to estimate the hazard ratio (HR) after adjusting for the selected variables. OS was measured from the time of primary surgery to the time of all-cause death, and for patients who did not die, was censored at the time of the last contact. OS distribution was estimated using the Kaplan–Meier method. Both log-rank test and Cox proportional hazards regression model were performed to test the difference in survival between groups. To accurately assess the clinical impact of adjuvant chemotherapy for survival, we investigated the OS and DFS using propensity score-matching method. For each participant, propensity score, the probability for receiving adjuvant chemotherapy given clinically important risk factors for DFS and OS, was estimated using a logistic regression model. Concretely, the adjusted risk factors are PS ≥ 2, comorbidity, ER, HER2, pT ≥ 2, pN ≥ 1, pStage ≥ 2, age, and BMI ≥ 24 (Supplementary Fig. 1). Patients were matched with a fixed ratio of 1:1 using the nearest neighbor within the caliper of 0.25 standard deviations. All tests were two-sided. The statistical significance was set at *P* < 0.05. All statistical analyses were conducted with JMP ver. 14 (SAS Institute Inc., Cary, NC, USA).

## Results

### Patient characteristics

**Fig. 1:**
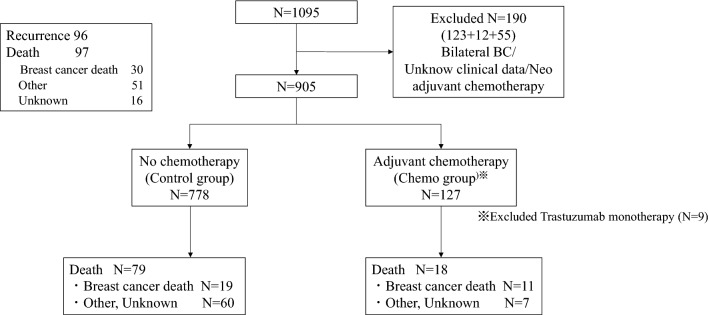
Consort diagram. Flow chart illustrating the number of study participants in each group

A total of 1095 older patients with breast cancer undergoing primary surgery were enrolled, and 905 patients [mean (SD) age, 75 (4.6) years] were included in the analyses. Of these, 127 patients (14%) received adjuvant chemotherapy (Chemo group), excluding trastuzumab monotherapy, and 778 (86%) did not (Control group) (Fig. 1). Patients’ characteristics among the two groups are listed in Table 1. Compared with the Control group, the Chemo group was younger (mean age 73 vs 76 years; *P* < 0.001), had a larger primary tumor size (mean size 25.9 vs 19.9 mm; *P* < 0.001), more metastatic lymph nodes (median 2.9 vs 0.7; *P* < 0.001), and a higher degree of the pathological stage. In addition, more patients were estrogen receptor (ER) negative or HER2 positive in the Chemo group. No difference in the type of primary breast surgery performed (total or partial mastectomy), PS status, body mass index (BMI), and presence of comorbidity between both groups were found.

**Table 1:** Clinicopathological characteristics

	Control group [*N* = 778 (%)]	Chemo group [*N* = 127 (%)]	*P* value
Objective periods, month (range)	70.6 (0.2–145)	79 (6.7–145.5)	0.0278
Mean age, year (range)	76 (70–93)	73 (70–87)	< 0.0001
pStage			
I	437 (56.2)	21 (16.5)	< 0.0001
II	301 (38.7)	77 (60.6)	
III	40 (5.1)	29 (22.8)	
Pathological tumor size, cm (range)	19.9 (1–80)	25.9 (0.5–100)	< 0.0001
Positive axillary lymph nodes (range)	0.7 (0–25)	2.9 (0–34)	< 0.0001
Pathological type			
Ductal carcinoma	707 (90.9)	121 (95.3)	0.2117
Other	71 (9.1)	6 (4.7)	
Estrogen receptor status			
Positive	682 (87.7)	68 (53.5)	< 0.0001
Negative	89 (11.4)	59 (46.5)	
Unknown	7 (0.9)	0	
HER2 status			
Positive	79 (10.2)	37 (29.1)	< 0.0001
Negative	642 (82.5)	88 (69.3)	
Unknown	57 (7.3)	2 (1.6)	
Ly			
0	556 (71.5)	56 (44.1)	< 0.0001
1	181 (23.3)	54 (42.5)	
2	23 (3.0)	14 (11.0)	
3	12 (1.5)	8 (2.4)	
Unknown	6 (0.7)	0	
pT			
T1	505 (64.9)	46 (36.2)	< 0.0001
T2	248 (31.9)	72 (56.7)	
T3	18 (2.3)	5 (3.9)	
T4	7 (0.9)	4 (3.2)	
pN			
N0	604 (78.2)	53 (41.7)	< 0.0001
N1	135 (17.5)	47 (37.0)	
N2	24 (3.1)	20 (15.8)	
N3	7 (0.9)	7 (5.5)	
Unknown	2 (0.3)	0	
Mean BMI (range)	23.2 (12.8–46.7)	23.5 (14.8–38.0)	0.3349
Comorbidity			
Present	568 (73.0)	94 (74.0)	0.8121
Absent	210 (27.0)	33 (26.0)	
ECOG performance status			
0	553 (71.1)	102 (80.3)	0.2070
1	184 (23.7)	22 (17.3)	
2 ≤	38 (4.9)	3 (2.4)	
Surgery			
Mastectomy	372 (47.8)	71 (55.9)	0.2252
Partial mastectomy	405 (52.2)	56 (44.1)	
Endocrine therapy			
Present	622 (80.0)	69 (54.3)	< 0.0001
Absent	155 (20.0)	58 (46.7)	

### Patient outcomes

**Fig. 2:**
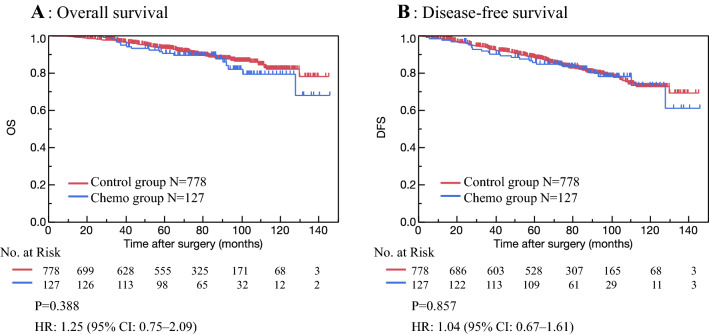
Overall survival (OS) and disease-free survival (DFS) in patients who received and did not receive adjuvant chemotherapy. OS and DFS rates were not significantly different in the Control and Chemo groups (**A** OS: *P* = 0.388; **B** DFS: *P* = 0.857). Tick marks indicate censored data

The median follow-up period was 70.6 (range 0.2–145) months in the Control, and 79.0 (range 6.7–145.5) months in the Chemo group (Table 1). OS and DFS are shown in Fig. 2. OS and DFS were 80.8% [95% confidence interval (CI) 73.9–86.7] and 77.8% [95% CI 73.5–85.3]; 74.4% [95% CI 73.1–77.0%] with adjuvant chemotherapy versus 73.5% [95% CI 72.3–74.9] without, respectively. There were no significant differences in OS (*P* = 0.388) and DFS (*P* = 0.857) between the two groups. The results of univariate analysis and multivariate Cox regression analyses for OS and DFS are shown in Tables 2 and 3. In the univariate analysis, PS ≥ 2, presence of comorbidity, ER negative, pT ≥ 2, pN ≥ 1, pStage ≥ 2, and older age were considered poor prognostic factors; however, chemotherapy did not affect OS or DFS. Similarly, in multivariate analysis, ER negative, pStage ≥ 2, and older age were considered as poor prognostic factors; in addition, chemotherapy did not improve prognosis.

**Table 2:** Univariate analysis of potential prognostic factors for overall and disease-free survival

Potential prognostic factor	Overall survival		Disease-free survival	
	HR (95% CI)	*P* value	HR (95% CI)	*P* value
Age	1.089 (1.042–1.137)	0.0003	1.068 (1.030–1.105)	0.0005
Comorbidity	1.854 (1.099–3.123)	0.0208	1.579 (1.648–2.341)	0.0231
PS ≥ 2	3.164 (1.327–6.362)	0.0122	2.420 (1.142–4.487)	0.0234
ER	0.526 (0.333–0.830)	0.0058	0.4523 (0.317–0.645)	< 0.0001
HER2	1.206 (0.682–2.133)	0.5189	1.024 (0.639–1.641)	0.9216
pT ≥ 2	2.634 (1.748–3.968)	< 0.0001	2.809 (2.028–3.893)	< 0.0001
pN ≥ 1	2.102 (1.398–3.159)	0.0004	2.111 (1.528–2.916)	< 0.0001
pStage ≥ 3	3.442 (2.060–5.752)	< 0.0001	3.859 (2.586–5.759)	< 0.0001
pStage ≥ 2	2.315 (1.510–3.548)	0.0001	2.478 (1.762–3.485)	< 0.0001
Ly ≥ 1	1.463 (0.972–2.203)	0.0683	1.729 (1.254–2.384)	0.0008
BMI ≥ 24	1.063 (0.709–1.595)	0.7673	1.061 (0.770–1.463)	0.7171
Chemotherapy	1.252 (0.750–2.090)	0.3894	1.041 (0.673–1.609)	0.8566

**Table 3:** Multivariate cox regression analyses for overall and disease-free survival

Potential prognostic factor	Overall survival		Disease-free survival	
	HR (95% CI)	*P* value	HR (95% CI)	*P* value
Age	1.077 (1.026–1.130)	0.0025	1.054 (1.015–1.094)	0.0067
Comorbidity	1.371 (0.801–2.348)	0.2495	1.262 (0.843–1.887)	0.2582
PS ≥ 2	2.046 (0.866–4.835)	0.1026	1.474 (0.708–3.072)	0.2998
ER	0.513 (0.308–0.853)	0.0100	0.372 (0.251–0.551)	< 0.0001
HER2	0.811 (0.442–1.490)	0.5006	0.676 (0.409–1.117)	0.1268
pStage ≥ 2	1.591 (0.790–3.201)	0.0207	2.151 (1.499–3.085)	< 0.0001
Chemotherapy	0.997 (0.544–1.825)	0.9917	0.645 (0.392–1.063)	0.0857

### Propensity score-matched analysis

**Fig. 3:**
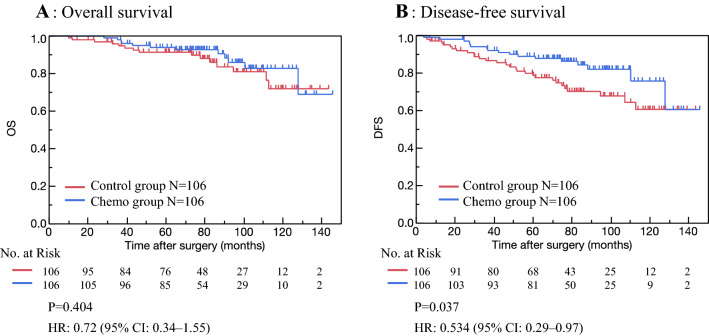
Overall survival (OS) and disease-free survival (DFS) in patients who received, and did not receive adjuvant chemotherapy after matching. In the matching analyses, OS was not significantly different between the Control and Chemo groups (*P* = 0.404). In contrast, DFS differed significantly between the two groups (*P* = 0.037). Tick marks indicate censored data

We evaluated whether chemotherapy affects prognosis after adjusting background factors with propensity score-matching. We checked that covariates were balanced across the Chemo and Control groups within strata of the propensity score (Supplementary Fig. 1). A total of 106 patients for each group were included in the matched analyses (Table 4). After matching, only PS was not adjusted. The median follow-up period was 77.3 (range 2–143.7) and 80.3 (range 6.7–145.5) months in the Control and Chemo groups, respectively. Kaplan–Meier curves for OS and DFS are shown in Fig. 3. DFS was found to be significantly prolonged with adjuvant chemotherapy (*P* = 0.037). However, there was no significant difference in the OS between the Control and the Chemo groups in the matched cohort (*P* = 0.404).

**Table 4:** Clinicopathologic characteristics of the Control and Chemo groups in the propensity score-matched analysis, before and after matching

Variable	All patients			Propensity-matched patients		
	Control group *N* = 782	Chemo group *N* = 184	*P* value	Control group *N* = 106	Chemo group *N* = 106	*P* value
Objective periods (month), mean (range)	70.6 (0.2–145)	79 (6.7–145.5)	0.0278	77.3 (2–143.7)	80.3 (6.7–145.5)	0.1276
Age (year), mean (range)	76 (70–93)	73 (70–87)	< 0.0001	73 (70–82)	73 (70–87)	0.8423
ECOG performance status, no (%)						
0	553 (71.1)	102 (80.3)	0.2070	71 (67.0)	87 (82.1)	0.0344
1	184 (23.7)	22 (17.3)		22 (31.1)	17 (16.0)	
2 ≥	38 (4.9)	3 (2.4)		2 (1.9)	2 (1.9)	
Pathologic stage, no (%)						
I	437 (56.2)	21 (16.5)	< 0.0001	18 (17.0)	21 (19.8)	0.8670
II	301 (38.7)	77 (60.6)		67 (63.2)	65 (61.3)	
III	40 (5.1)	29 (22.8)		21 (19.8)	20 (18.9)	
Pathological tumor size (mm), mean (range)	19.9 (1–80)	25.9 (0.5–100)	< 0.0001	23 (0.2–76)	22 (1–80)	0.8471
Positive axillary lymph nodes, median (range)	0.7 (0–25)	2.9 (0–34)	< 0.0001	1.8 (0–15)	2.6 (0–34)	0.2022
Pathological type, no (%)						
Ductal carcinoma	707 (90.9)	121 (95.3)	0.2117	99 (93.4)	101 (95.3)	0.5522
Other	71 (9.1)	6 (4.7)		7 (6.6)	5 (4.7)	
Estrogen receptor status, no (%)						
Positive	682 (87.7)	68 (53.5)	< 0.0001	39 (36.8)	43 (40.6)	0.5727
Negative	89 (11.4)	59 (46.5)		67 (63.2)	63 (59.4)	
Unknown	7 (0.9)	0		0	0	
HER2 status, no (%)						
Positive	79 (10.2)	37 (29.1)	< 0.0001	80 (75.5)	76 (71.7)	0.5332
Negative	642 (82.5)	88 (69.3)		26 (24.5)	30 (28.3)	
Unknown	57 (7.3)	2 (1.6)		0	0	
Ly, no (%)						
0	556 (71.5)	56 (44.1)	< 0.0001	57 (53.8)	47 (44.4)	0.5315
1	181 (23.3)	54 (42.5)		37 (34.9)	46 (43.4)	
2	23 (3.0)	14 (11.0)		8 (7.6)	10 (9.4)	
3	12 (1.5)	8 (2.4)		3 (2.8)	3 (2.8)	
Unknown	6 (0.7)	0		1 (0.9)	0

## Discussion

This study was a large retrospective cohort of older patients with breast cancer from Japan using real-world data. We investigated whether adjuvant chemotherapy is needed for older patients with breast cancer. Older patients with breast cancer are a heterogeneous group with multiple comorbidities and functional decline. Thus, it is difficult for them to be included in the traditional randomized controlled trial [8].

We found that patients who received adjuvant chemotherapy had larger primary tumor size, many numbers of metastatic lymph nodes, and a higher degree of pathological stage (Table 1). Adjuvant chemotherapy is recommended in patients with high numbers of involved lymph nodes and large tumor sizes. There were no significant differences in OS and DFS between the Chemo and Control groups (Fig. 2), which suggested that adjuvant chemotherapy might have improved the prognosis. Previously, Tamirisa et al. [9] found that adjuvant chemotherapy was associated with improved survival in lymph node-positive, ER-positive older patients with comorbidities. Derks et al. [2] showed that breast cancer mortality was higher and ratios of chemotherapy were lower in ER-positive patients without comorbidity older than 70 years. Elkin et al. [10] found a survival benefit in lymph node-positive and ER-negative patients who received chemotherapy. Our results showed that adjuvant chemotherapy improved DFS after adjusting with variations in patient characteristics (Fig. 3). Chemotherapy plays an important role in older patients, but it is found to be associated with adverse events.

Among 127 patients receiving chemotherapy, results showed that 46 (36.2%) patients experienced adverse events and 54 (42.5%) reduced or discontinued chemotherapy. There was no chemotherapy-related death reported, similar to previous studies [11, 12]. Our results showed that adjuvant chemotherapy did not improve OS in the matching cohort (Fig. 3). There may be limited survival benefits and increased risk of toxicities in older patients. Because older patients have heterogeneity in health status and limited prognosis, it is important to consider their background before deciding the type of treatment [2, 13].

Recently, the therapeutic approach has shifted from classical chemotherapy toward targeted therapies (i.e., anti-HER2 blocker, CDK4/6 inhibitor) [14]. The RESPECT trial is a randomized adjuvant trial comparing trastuzumab monotherapy with trastuzumab plus chemotherapy for HER2-positive older patients with breast cancer [15, 16]. Although the primary endpoint was not met, trastuzumab monotherapy could be considered an adjuvant therapy option for selected older patients. CDK4/6 inhibitors are an attractive option for older patients with advanced ER-positive, and HER2-negative breast cancer [17]. Abemaciclib combined with endocrine therapy demonstrated a significant improvement in invasive disease-free survival (IDFS) [18]. Furthermore, patients with germline *BRCA1/2* mutation (gBRCAm) are recommended for targeted and individualized cancer prevention and treatment [19]. Olaparib, a poly (adenosine diphosphate-ribose) polymerase (PARP) inhibitor which is used as a targeted therapy for gBRCAm, provided a significant benefit over standard therapy among patients with HER2-negative breast cancer, regardless of age [20, 21]. Women with gBRCAm typically develop breast cancer at an early age. Although this mutation decreases in older people, it is present in a certain population of older patients [22]. Abemaciclib and Olaparib are given orally and are generally well-tolerated [23]; thus, it is acceptable as a treatment for older patients. Considering the mechanism of action, it might be beneficial in targeted therapies without chemotherapy, and possible to develop less toxic treatment strategies, without chemotherapy for older patients. This study did not evaluate this aspect because it included patients from 2008 to 2013, none of whom received these molecular targeted drugs, and a small number of whom received trastuzumab alone. As previous studies with such drugs also had a lesser older patient population, further studies are needed.

This study has some limitations. It was retrospective in nature, for which we used a propensity score-matched analysis and adjusted background factors in an attempt to minimize selection bias. It should be noted that the backgrounds of the Chemo and Control groups had different characteristics (Supplementary Fig. 2). After matching, about 40% of patients were ER-positive and more than 70% were HER2-positive, which is quite different from the general distribution of subtype (Table 4). Additionally, we were unable to obtain a comprehensive geriatric assessment for PS, comorbidities, and adverse events of chemotherapy. Available data did not allow for the exploration of the relative contribution of these limitations to our results. Furthermore, we evaluated pathological factors excluding neoadjuvant chemotherapy, which was once reserved to reduce the size and extent of locally advanced tumors but is now being used more widely, due to its increased likelihood of tumor control, and potential for curability in early breast cancer [24, 25]. Therefore, it is now necessary to investigate effective drugs and the efficacy of neoadjuvant chemotherapy in older patients. Finally, chemotherapeutic regimens were determined by the physician’s choice at that time. With time, treatments have advanced, such as molecular targeted therapy; thus, the results should be interpreted with caution.

In conclusion, the results showed that adjuvant chemotherapy could not improve the overall survival of older patients with breast cancer after propensity score matching. Limited data are available on the benefit of chemotherapy in older patients with breast cancer. More research is needed to determine the use of neoadjuvant and adjuvant chemotherapy in older patients; therefore, we need to conduct prospective studies on the efficacy of chemotherapy in the near future.

## Supplementary Information

Below is the link to the electronic supplementary material.

Supplementary file1 (DOCX 106 KB)

## Data Availability

The datasets during and/or analyzed during the current study are available from the corresponding author on reasonable request.
