# Supplementary material for: Is adjuvant chemotherapy necessary in older patients with breast cancer?
Source: Breast Cancer. 2022 Jan 15;29(3):498–506. doi: 10.1007/s12282-021-01329-7 (PMC9021076; doi:10.1007/s12282-021-01329-7)
Supplement: Supplementary file 1 — Supplementary file1 (DOCX 106 KB) [file 12282_2021_1329_MOESM1_ESM.docx]

**Supplementary Material**

Supplementary Figure 1. ROC curve of propensity scores.

Supplementary Figure 2. Propensity score distribution in the Control and Chemo groups before propensity score matching.

Supplementary Figure 1. ROC curve of propensity scores.

The adjusted risk factors are PS ≥2, comorbidity, ER, HER2, pT≥2, pN≥1, pStage≥2, age, and BMI≥24.

Sensitivity

1-Specificity

Supplementary Figure 2. Propensity score distribution in the Control and Chemo groups before propensity score matching.

The distribution is different in each group.

Control group Chemo group
